# Analysis of the association between reading and writing postures and comorbidity of myopia and scoliosis in junior middle school students

**DOI:** 10.3389/fped.2025.1576575

**Published:** 2025-09-18

**Authors:** Yanjiao Wang, Dongling Yang, Fengyun Zhang, Wenjuan Qi, Qiuying Lu, Haotian Wu, Chunyan Luo

**Affiliations:** ^1^ Division of Child and Adolescent Health, Shanghai Municipal Center for Disease Control and Prevention, Shanghai, China; ^2^ School of Public Health, Shanghai University of Traditional Chinese Medicine, Shanghai, China; ^3^ Shanghai Eye Disease Prevention and Treatment Center, Shanghai Eye Hospital, Shanghai, China

**Keywords:** myopia, scoliosis, adolescent, poor reading/writing postures, comorbidity

## Abstract

**Objective:**

Given the emerging co-prevalence of myopia and scoliosis as significant public health challenges among Chinese adolescents, and considering sustained poor reading/writing postures as a potential shared risk factor contributing to onset, progression, and comorbidity, this study aimed to investigate the epidemiological association between myopia and scoliosis, specifically evaluate the synergistic effects of poor reading/writing postures on these conditions, and establish evidence-based strategies for coordinated prevention of comorbid orthopaedic-ophthalmic disorders.

**Methods:**

The study population comprised adolescents aged 11–15 years enrolled in junior middle schools across Shanghai. All participants underwent comprehensive ocular examinations, standardized scoliosis screening, and completed structured questionnaires assessing demographic and behavioral risk factors.

**Results:**

This study included 9,583 middle school students (mean age 12.59 ± 1.17 years). Overall myopia prevalence was 77.6%, while the scoliosis prevalence was 1.7%. Notably, 87.2% of the scoliosis cohort had concurrent myopia, compared to 77.4% in the non-scoliosis group. The dual-disease comorbidity rate was 1.5% (males: 1.0%; females: 2.1%). Multivariable logistic regression analysis revealed that poor reading/writing postures (OR = 1.17, 95% CI: 1.02–1.34) and scoliosis screening positivity (OR = 1.74, 95% CI: 1.09–2.76) were significantly associated with increased myopia risk. Conversely, myopia demonstrated a bidirectional association with elevated scoliosis susceptibility (OR = 1.73, 95% CI: 1.09–2.75). For dual-disease cases, advancing school grade and female sex were positively correlated with comorbidity. Systematic implementation of postural breaks after 30-minute near-work intervals (OR = 0.65, 95% CI: 0.46–0.91) and teacher-mediated posture monitoring (OR = 0.66, 95% CI: 0.45–0.97) significantly reduced comorbidity risks compared to sporadic practice.

**Conclusions:**

A significant bidirectional association exists between adolescent myopia and scoliosis, with non-ergonomic reading/writing postures identified as a shared modifiable risk factor. Education functional departments should implement evidence-based interventions including postural ergonomics education, routine vision and spinal screening programs, and structured postural breaks after near-work intervals to mitigate dual-disease burdens in adolescents.

## Introduction

Emerging evidence suggests thatpoor reading/writing postures during near-work activities may concurrently drive the development of myopia and adolescent idiopathic scoliosis (AIS)—two ostensibly distinct yet mechanistically interconnected health burdens in Chinese adolescents during the critical transition to middle school (1). This dual-disease paradigm warrants urgent investigation given their shared modifiable risk factors and peak susceptibility coinciding with pubertal development.

Myopia, the most prevalent refractive error, is primarily characterized by excessive elongation of the ocular axis (2–5). In recent years, the incidence of myopia among children and adolescents has risen annually due to increasing academic burden and frequent use of electronic devices, evolving into a significant global public health challenge (6). Studies project that approximately 100 million individuals in China may suffer irreversible vision impairment or blindness from myopia by 2050, imposing substantial socioeconomic burdens (7). The etiology of myopia is multifactorial, with extensive research highlighting genetic predisposition and environmental factors as key contributors (8–11). Among these, modifiable environmental factors—such as academic burden (12, 13), insufficient outdoor activity (14, 15), prolonged electronic device usage (16, 17), inadequate sleep duration (18–20), poor reading/writing postures (21, 22), and prolonged near work (23, 24)—play a critical role in prevention strategies. Critically, suboptimal postures during near-work exacerbate ocular axial elongation by inducing retinal defocus and dopamine deficiency, while simultaneously imposing asymmetric spinal loads. Notably, the synergistic impact of these factors may be amplified during early adolescence, a developmental stage characterized by rapid skeletal growth and ocular axial elongation prior to peak puberty (25–29).

Notably, poor reading/writing postures have emerged as a focal concern for adolescent health, with biomechanical and neurophysiological links to spinal deformity. Biomechanically, sustained trunk flexion and lateral bending during desk work generate uneven pressure on vertebral growth plates, potentially accelerating AIS progression through asymmetric neurocentral junction activity (30). Neurophysiologically, impaired glycinergic neurotransmission—recently implicated in AIS pathogenesis due to its role in coordinating paraspinal muscle symmetry—may be aggravated by postural stress-induced neural pathway dysregulation (31).

Scoliosis, a prevalent spinal disorder among adolescents in China, has been reported to affect 0.11%–2.64% of this population (32). This condition not only alters body appearance and limb symmetry but may also severely impair motor coordination and cardiopulmonary functions in advanced cases. Without timely diagnosis and intervention, progressive deformity may lead to permanent disability, causing substantial physical and psychological harm to adolescents (33–35). Importantly, clinical studies report a 1.49-fold higher incidence of myopia in AIS patients vs. controls, suggesting shared biomechanical triggers from postural habits (36).

In response, the Chinese government integrated scoliosis and myopia prevention into the 2018 National Student Common Diseases and Health Risk Factors Surveillance Program (37), explicitly targeting posture correction as a dual-disease intervention. This study investigates the association between adolescent myopia and scoliosis, with particular emphasis on the synergistic effects of poor reading/writing postures. The findings aim to inform evidence-based health policy formulation and promote effective adolescent health strategies.

## Methods

### Study setting and participant selection

**Figure 1:**
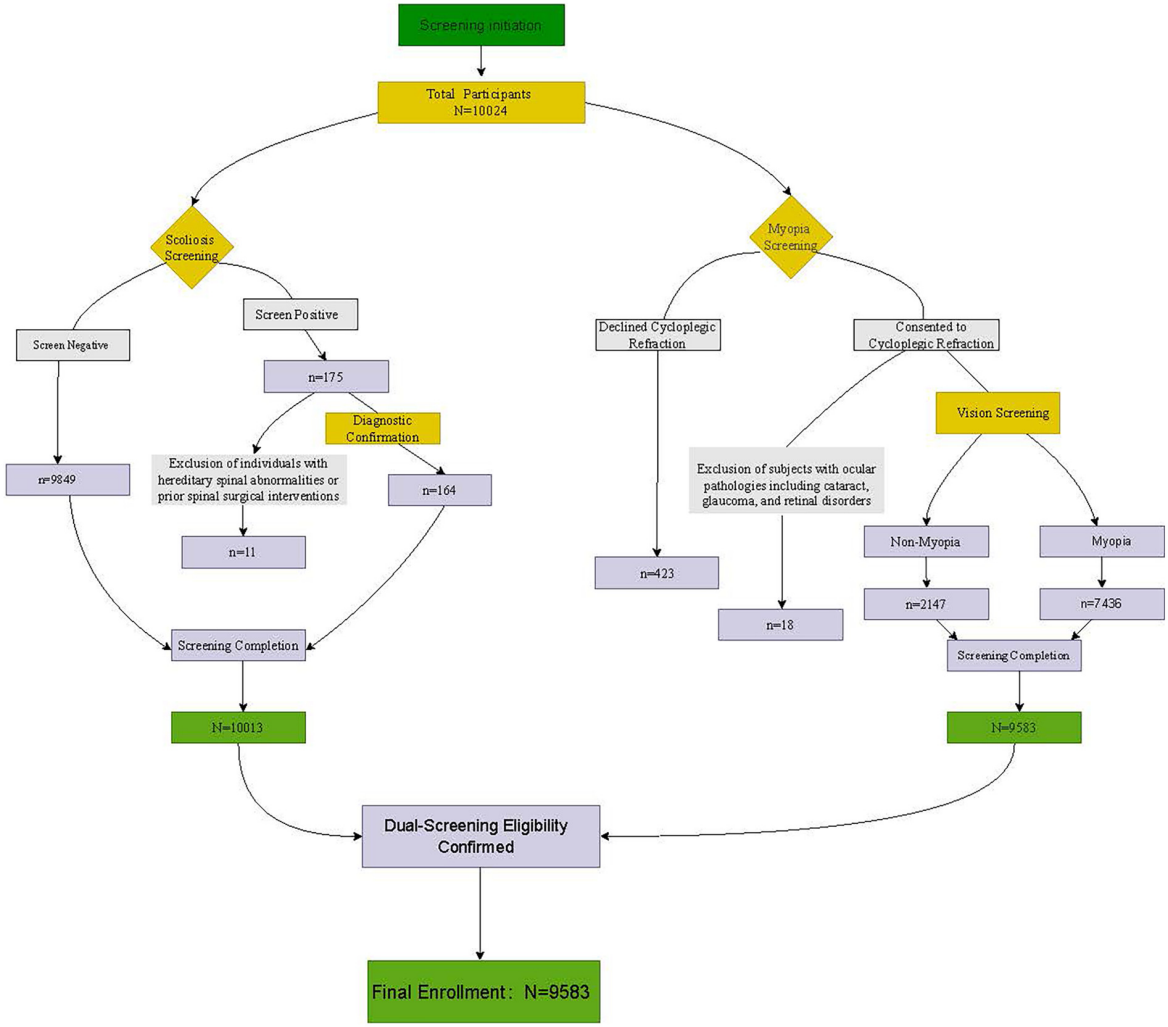
Screening protocol flowchart.

Based on the Student Common Diseases and Health Influencing Factors Surveillance Program conducted in Shanghai from October to November 2023, this study employed a cluster random sampling method. All public junior high schools across the city's 16 administrative districts were assigned unique identification codes. Subsequently, two schools were randomly selected from each district, resulting in a final sample of 32 schools. Data from 9,583 students enrolled in preparatory classes (Grade 6) and Grades 1 to 3 of junior middle school were included in the analysis. In Shanghai's educational structure where Grade 6 constitutes the preparatory phase integrated into junior middle schools, this initial secondary year serves to acclimate students (ages 11–12) to the secondary academic environment. The deliberate inclusion of this specific Grade 6 cohort captures the critical window of pubertal onset (Tanner stages II–III), where rapid skeletal growth and ocular axial elongation converge (38, 39). Targeting students at this precise transition point into secondary school is crucial as, while puberty staging was not directly measured, their age range aligns with early adolescence, where posture-mediated biomechanical stress may disproportionately impact comorbidity risk. Therefore, this study included students across Grades 6 through 9 for unified analysis. All participants provided written informed consent from either themselves or their legal guardians prior to enrollment and completed standardized questionnaires. Participants underwent visual acuity assessments and scoliosis screenings, while individuals with ocular pathologies (e.g., cataract, glaucoma, retinal disease) or hereditary spinal abnormalities were excluded. The study was conducted in accordance with the principles of the Declaration of Helsinki. The inspection flowchart is shown in Figure 1.

### Vision screening

In each school, licensed ophthalmologists and certified optometrists performed monocular distance visual acuity testing followed by comprehensive autorefractometry (Topcon KR-8900, Tokyo, Japan), strictly adhering to national myopia screening protocols (40). The autorefractometer was calibrated using artificial eyes with cylindrical lenses set to negative mode. Three consecutive measurements per eye were averaged to derive spherical equivalent (SE) values (to two decimal places). Re-testing was performed if inter-measurement variability exceeded 0.50 D to ensure data integrity.

### Questionnaire administration

A standardized questionnaire from the Shanghai Municipal Common Disease Surveillance Initiative (Student Health Status and Influencing Factors Inventory, Junior Middle School Edition) (40) was administered via electronic data capture (EDC) systems. Data included:

1. Demographics: Grade (6–9), sex (male/female)
2. Health outcomes: Myopia (yes/no), scoliosis (yes/no), comorbidity status
3. Reading/writing posture behaviors (frequency: never, sometimes, usually, always):
  Keep the chest more than one fist-width from the edge of the desk (≈10 cm);
  Position eyes over one Chinese foot from the bookt (≈33 cm);
  Hold the pen with fingers about one Chinese inch from the pen tip (≈3.3 cm).
  Postural breaks after 30 min near-work;
4. Environmental factors (frequency as above):
  Teachers reminded to pay attention to reading and writing posture;
  Parental reminded to pay attention to reading and writing posture.

### Scoliosis screening

Screening personnel comprised licensed general practitioners and rehabilitation physicians from community health centers. All examiners completed standardized training and competency assessments. Procedures followed national guidelines (GB/T 16133-2014) (41):

1. General inspection: Participants stood naturally with feet shoulder-width apart. Posterior view assessment included shoulder height symmetry, scapular alignment (inferior angle levelness), lumbar contour symmetry, iliac crest levelness, and spinal process deviation.
2. Adams forward bend test: Forward bending with arms extended. Observed asymmetry (e.g., unilateral rib prominence, muscle tightness) was recorded as positive, prompting scoliometer measurement of the angle of trunk rotation (ATR).
3. Supplemental mobility assessment: Performed if postural asymmetry was suspected during general inspection despite negative bend test.

## Judgment criteria

### Myopia

Spherical equivalent (SE) was calculated as spherical power plus half of the cylindrical power (SE = spherical + cylindrical/2) (40). Myopia was diagnosed when cycloplegic autorefraction revealed SE <−0.50 diopters (D). For participants with interocular differences, the diagnosis was based on the eye with worse visual acuity.

### Poor reading/writing posture

Postural habits were assessed based on three criteria during reading/writing activities: Chest-to-desk distance exceeding a fist's width (≤10 cm), Eye-to-book distance exceeding one Chinese foot (≈33 cm), Finger-to-pen-tip distance deviating from one Chinese inch (≈3.3 cm),Postural breaks after 30 min near-work. Participants responding “frequently” or “always” to all four items were classified as having appropriate posture (42). Those reporting “never” or “Sometimes” for any item were categorized as having poor posture.

### Scoliosis grading criteria

No scoliosis: No abnormalities detected on general inspection and negative forward bend test with axial trunk rotation (ATR) <5°.

Grade I: Abnormal general inspection or positive forward bend test or ATR ≥5°, followed by trunk rotation inclinometer measurement post spinal mobility assessment showing 5° ≤ ATR < 7°.

Grade II: Abnormal general inspection or positive forward bend test or ATR ≥5°, with post-assessment inclinometer measurement yielding 7° ≤ ATR < 10°.

Grade III: Abnormal general inspection or positive forward bend test or ATR ≥5°, confirmed by post-assessment inclinometer measurement with ATR ≥10° (43).

### Comorbidity of myopia and scoliosis

Individuals diagnosed with both scoliosis and myopia were classified as having comorbidity, reflecting the co-occurrence of these conditions. In contrast, those demonstrating normal visual acuity (spherical equivalent ≥−0.50 D) without spinal curvature abnormalities (ATR <5° and negative forward bend test) were categorized as the non-comorbid group.

### Statistical analysis

Survey data were double-entered into the EpiData 3.1 database to ensure accuracy. Statistical analyses were performed using SPSS version 26.0. Categorical variables are expressed as numbers and percentages, while continuous variables are presented as mean ± standard deviation. Multivariate logistic regression models were employed to identify factors associated with myopia, scoliosis, and their comorbidity, adjusting for covariates including grade level, sex, and other potential confounders. A *P*-value <0.05 was considered statistically significant.

## Results

**Figure 2:**
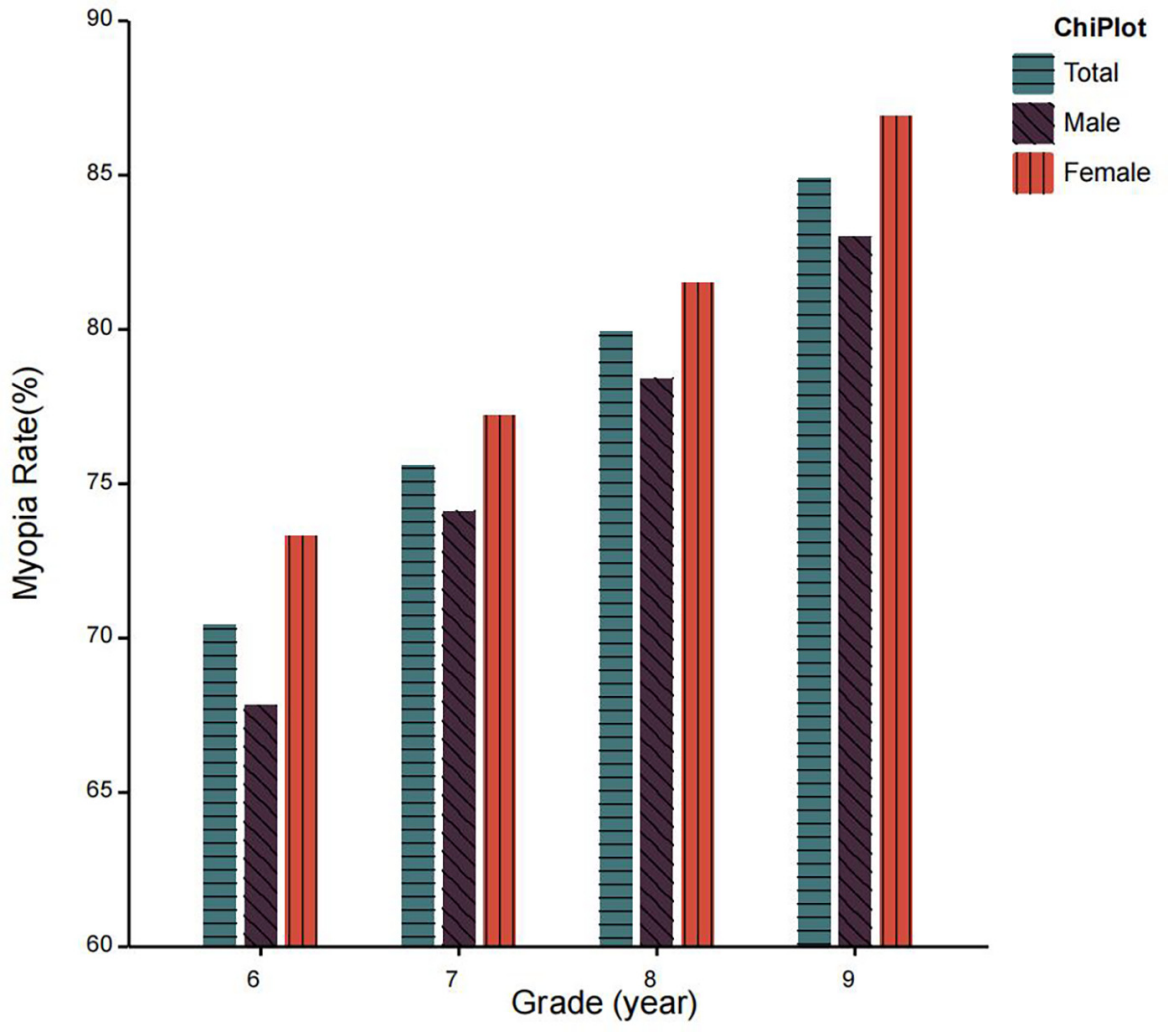
Prevalence of myopia in different grades.

**Figure 3:**
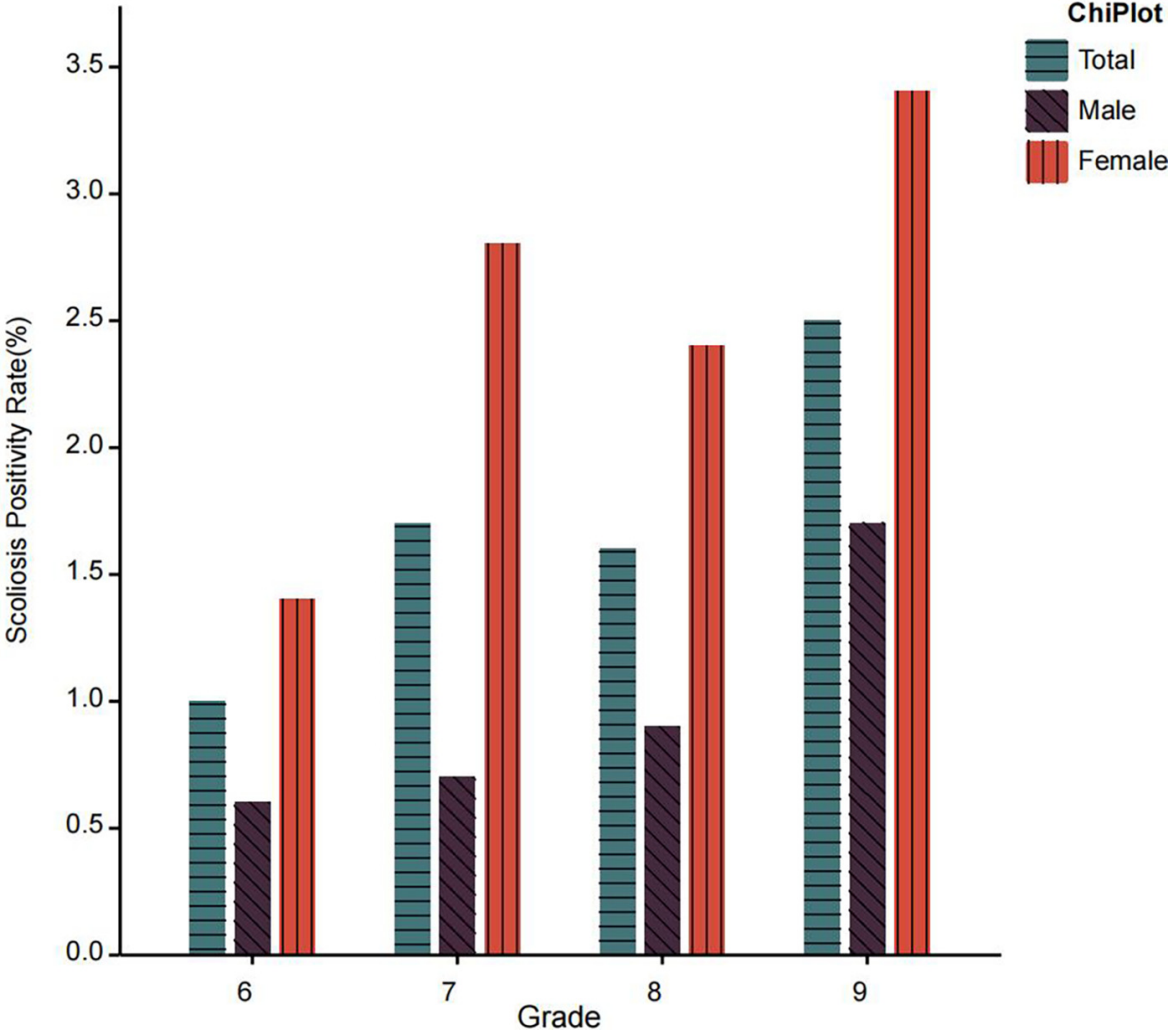
Prevalence of scoliosis in different grades.

The study included a total of 9,583 junior middle school students, comprising 4,935 males and 4,648 females. Participants were distributed across grades 6–9 with 2,459, 2,361, 2,498, and 2,265 students in each respective grade. The mean age of the cohort was 12.58 ± 1.16 years. The overall prevalence of myopia in the population was 77.6%, with sex-specific rates of 75.7% in males and 79.6% in females (Figure 2). The scoliosis prevalence rate was 1.7% overall, demonstrating significant sex differences (1.0% in males vs. 2.5% in females). Comorbidity analysis revealed that 1.5% of the total population exhibited both conditions concurrently, with distinct sex-based variations in comorbidity rates (1.0% in males vs. 2.1% in females) (Figure 3). Disease characteristics stratified by gender are presented in Table 1. Behavioral analysis revealed: 57.1% maintained a fist-width distance between chest and desk edge (males 57.3% vs. females 56.9%); 63.7% kept fingers 3.3 cm from pen tip (males 63.0% vs. females 64.4%); only 39.4% rested after 30 min of continuous eye use (males 41.8% vs. females 36.8%). Compliance rates for posture reminders from teachers and parents were 45.7% and 64.5%, respectively. All percentages were calculated based on subgroup sample sizes and rounded to one decimal place, with data consistency verified. The characteristics of poor reading and writing postures stratified by gender are presented in Table 2.

**Table 1:** Characteristics of the study population.

Variable	Total (*n* = 9,583)	Male (*n* = 4,935)	Female (*n* = 4,648)
Grade distribution, *n* (%)			
Grade 6	2,459 (25.7%)	1,285 (26.0%)	1,174 (25.3%)
Grade 7	2,361 (24.6%)	1,204 (24.4%)	1,157 (24.9%)
Grade 8	2,498 (26.1%)	1,271 (25.8%)	1,227 (26.4%)
Grade 9	2,265 (23.6%)	1,175 (23.8%)	1,090 (23.5%)
Age (years), mean ± SD	12.58 ± 1.16	12.58 ± 1.17	12.58 ± 1.15
Myopia prevalence, *n* (%)	7,436 (77.6%)	3,735 (75.7%)	3,701 (79.6%)
Scoliosis prevalence, *n* (%)	164 (1.7%)	49 (1.0%)	115 (2.5%)
Myopia and scoliosis comorbidity, *n* (%)	143 (1.5%)	45 (0.9%)	98 (2.1%)

**Table 2:** Influential factors on the development of myopia and scoliosis, *n*, (%).

Characteristics	Total (*n* = 9,583)	Male (*n* = 4,935)	Female (*n* = 4,648)
The chest more than the width of a fist from the edge of the table			
Never and Sometimes	4,109 (42.9%)	2,107 (42.7%)	2,002 (43.1%)
Usually and Always	5,474 (57.1%)	2,828 (57.3%)	2,646 (56.9%)
The eyes are more than 33 cm away from the book			
Never and Sometimes	4,037 (42.1%)	2,046 (41.5%)	1,991 (42.8%)
Usually and Always	5,546 (57.9%)	2,889 (58.5%)	2,657 (57.2%)
The finger about 3.3 cm away from the pen tip			
Never and Sometimes	3,480 (36.3%)	1,825 (37.0%)	1,655 (35.6%)
Usually and Always	6,103 (63.7%)	3,110 (63.0%)	2,993 (64.4%)
Postural breaks after 30 min near-work			
Never and Sometimes	5,810 (60.6%)	2,871 (58.2%)	2,939 (63.2%)
Usually and Always	3,773 (39.4%)	2,064 (41.8%)	1,709 (36.8%)
Teachers reminded to pay attention to reading and writing postures			
Never and Sometimes	5,202 (54.3%)	2,582 (52.3%)	2,620 (56.4%)
Usually and Always	4,381 (45.7%)	2,353 (47.7%)	2,028 (43.6%)
Parents reminded to pay attention to reading and writing postures			
Never and Sometimes	3,404 (35.5%)	1,679 (34.0%)	1,725 (37.1%)
Usually and Always	6,179 (64.5%)	3,256 (66.0%)	2,923 (62.9%)

Multivariable analysis demonstrated a graded increase in myopia risk with higher grade levels: compared to 6th graders, students in grade 7 (OR = 1.30, 95% CI: 1.15–1.48), grade 8 (OR = 1.70, 95% CI: 1.49–1.94), and grade 9 (OR = 2.40, 95% CI: 2.08–2.78) showed progressively elevated risks (*P*-trend < 0.001), with scoliosis risk peaking in grade 9 (OR = 2.40, 95% CI: 1.49–3.88). Females exhibited higher susceptibility to both myopia (OR = 1.24, 95% CI: 1.13–1.37) and scoliosis (OR = 2.42, 95% CI: 1.72–3.39). Critically, a bidirectional relationship was observed: scoliosis patients had 74% higher myopia risk (OR = 1.74, 95% CI: 1.09–2.76), while myopic individuals had 73% increased scoliosis risk (OR = 1.73, 95% CI: 1.09–2.75). Among posture-related factors, short finger-to-pen-tip distance (≤3.3 cm) increased myopia risk by 17% (OR = 1.17, 95% CI: 1.02–1.34), whereas parental posture reminders were paradoxically associated with higher myopia risk (OR = 1.14, 95% CI: 1.01–1.29), and reading distance >33 cm exerted protective effects (OR = 0.82, 95% CI: 0.70–0.95). For scoliosis, regular postural breaks (OR = 0.65, 0.46–0.91) and teacher posture reminders (OR = 0.66, 95% CI: 0.45–0.97) reduced risks by 35% and 34% respectively, while chest-to-desk distance and pen-holding behavior showed no significant associations (*P* > 0.05). as detailed in Table 3.

**Table 3:** Analysis of factors influencing myopia and scoliosis.

Characteristics	Myopia		Scoliosis	
	OR (95% CI)	*P*	OR (95% CI)	*P*
Grade				
6	1			
7	1.30 (1.15, 1.48)	<0.001	1.68 (1.02, 2.78)	0.04
8	1.70 (1.49, 1.94)	<0.001	1.56 (0.95, 2.59)	0.08
9	2.40 (2.08, 2.78)	<0.001	2.40 (1.49, 3.88)	<0.001
Sex				
Male	1			
Female	1.24 (1.13, 1.37)	<0.001	2.42 (1.72, 3.39)	<0.001
Scoliosis				
Not	1			
Yes	1.74 (1.09, 2.76)	0.02		
Myopia				
Not			1	
Yes			1.73 (1.09, 2.75)	0.02
The chest more thanthe width of a fist from the edge of the table				
Never and Sometimes	1			
Usually and Always	1.06 (0.92, 1.23)	0.40	0.98 (0.61, 1.56)	0.92
The eyes are more than 33 cm away from the book				
Never and Sometimes	1			
Usually and Always	0.82 (0.70,0.95)	0.01	1.32 (0.80,2.18)	0.27
The finger about 3.3 cm away from the pen tip				
Never and Sometimes	1			
Usually and Always	1.17 (1.02, 1.34)	0.03	0.79 (0.50, 1.24)	0.31
Postural breaks after 30 min near-work				
Never and Sometimes	1			
Usually and Always	0.92 (0.84, 1.02)	0.12	0.65 (0.46, 0.91)	0.01
Teachers reminded to pay attention to reading and writing posture				
Never and Sometimes	1			
Usually and Always	0.93 (0.82, 1.04)	0.21	0.66 (0.45, 0.97)	0.04
Parents reminded to pay attention to reading and writing postures				
Never and Sometimes	1			
Usually and Always	1.14 (1.01, 1.29)	0.04	1.09 (0.75, 1.58)	0.66

Previous studies have established poor posture as a contributing factor to myopia and scoliosis development in adolescents. Our study extends this evidence by systematically investigating the “co-morbidity, co-risk factors, and co-preventive strategies” underlying these prevalent conditions in this population. We implemented a novel analytical framework that categorized students with both conditions into a distinct comorbidity group and similarly grouped unaffected individuals. The findings revealed a significant escalation in the risk of comorbidity in tandem with academic progression, with odds ratios (ORs) for 7th, 8th, and 9th graders calculated at 1.73 (95% CI: 1.02–2.95, *p* = 0.04), 2.10 (95% CI: 1.23–3.57, *p* = 0.01), and 4.53 (95% CI: 2.72–7.53, *p* < 0.001). Female students exhibited significantly higher comorbidity risk compared to males (OR = 2.8, 95% CI: 1.94–4.05, *p* < 0.001). Notably, regular eye-rest intervals following 30 min of sustained near work emerged as a protective factor against comorbidity (OR = 0.63, 95% CI: 0.44–0.92, *p* = 0.02). Similarly, active teacher interventions to correct reading/writing postures were associated with reduced comorbidity risk (OR = 0.57, 95% CI: 0.37–0.88, *p* = 0.01). These findings are comprehensively summarized in Table 4.

**Table 4:** Analysis of factors influencing the co-morbidity of myopia and scoliosis.

Characteristics	*β*-value	OR-value	OR (95% CI)	*P*
Grade				
6	1			
7	0.549	1.73	(1.02, 2.95)	0.04
8	0.740	2.10	(1.23, 3.57)	0.01
9	1.510	4.53	(2.72, 7.53)	<0.001
Sex				
Male	1			
Female	1.029	2.80	(1.94, 4.05)	<0.001
The chest more than the width of a fist from the edge of the table				
Never and Sometimes	1			
Usually and Always	0.008	1.01	(0.60, 1.71)	0.98
The eyes are more than 33 cm away from the book				
Never and Sometimes	1			
Usually and Always	0.053	1.05	(0.60, 1.85)	0.86
The finger about 3.3 cm away from the pen tip				
Never and Sometimes	1			
Usually and Always	−0.107	0.90	(0.55, 1.47)	0.67
Postural breaks after 30 min near-work				
Never and Sometimes	1			
Usually and Always	−0.458	0.63	(0.44, 0.92)	0.02
Teachers reminded to pay attention to reading and writing posture				
Never and Sometimes	1			
Usually and Always	−0.561	0.57	(0.37, 0.88)	0.01
Parents reminded to pay attention to reading and writing postures				
Never and Sometimes	1			
Usually and Always	0.171	1.19	(0.78, 1.80)	0.42

## Discussion

This study presents an examination of the prevalence rates of myopia and scoliosis among junior middle school students in Shanghai, with a particular focus on the impact of suboptimal reading and writing postures on the development of myopia. Crucially, by explicitly incorporating the analysis of co-morbidity between these two prevalent conditions, our findings contribute significantly to the emerging “co-morbidity-co-causes and co-prevention” paradigm for adolescent health issues. This paradigm posits that shared behavioral and environmental risk factors, notably sustained poor posture during academic activities, may underlie the development of multiple co-occurring conditions (44–46). Our detailed investigation into specific reading and writing postures provides concrete evidence advancing this framework and lays a foundation for elucidating the underlying shared pathophysiological mechanisms.

Research has confirmed that high-intensity near work and poor reading/writing postures are significant risk factors for the development and progression of myopia. Notably, the prevalence of myopia is consistently higher among female students across all educational stages compared to males, a disparity potentially attributable to females' longer average duration of near work and relatively less time spent in outdoor activities (47, 48). Maintaining a reading distance greater than 33 cm has been established by multiple studies as a key protective factor against myopia (49–53). Concurrently, individuals screening positive for scoliosis exhibit a significantly higher risk of developing myopia compared to those without scoliosis. The prevalence of scoliosis among adolescents in China is approximately 1.8%, Given that scoliosis typically progresses rapidly during the adolescent growth spurt, timely intervention is crucial. Without intervention, the consequences extend beyond postural deformities to potentially include impaired cardiopulmonary function, posing long-term adverse effects on adolescents' overall health (54–59).

Findings further reveal that students with a positive scoliosis screening are more prone to adopting detrimental reading and writing postures. These poor postures not only significantly elevate the risk of myopia onset and progression but, if sustained long-term, can also exacerbate the advancement of scoliosis itself (60, 61). Importantly, individuals with myopia also demonstrate a heightened susceptibility to developing scoliosis. The underlying mechanism for this myopia-scoliosis comorbidity appears to stem from dysregulation within the “Visual-Vestibular-Postural Axis” (VVPA), Prolonged near-fixation induces intense ocular accommodation and convergence responses, creating a sustained demand on the visual system. This heightened visual demand can subconsciously trigger compensatory neuromuscular adaptations, including a forward head posture and spinal misalignment. Critically, this maladaptive posture disrupts proprioceptive feedback and vestibular function, potentially leading to altered muscle tone and asymmetrical loading on the developing spine. This creates a self-perpetuating cycle where the maladaptive posture facilitates continued myopia progression (e.g., by altering retinal defocus patterns) and simultaneously increases mechanical stress on the spine, thereby precipitating or exacerbating scoliosis (62). Consequently, Therefore, comprehensive interventions targeting the shared mechanisms of myopia and scoliosis hold significant potential for joint prevention and management. Correcting poor reading and writing postures is a core strategy, but effective intervention requires a multi-dimensional approach. At the school level, ergonomic education should be implemented, focusing on correct desk and chair height adjustment and regular posture correction. This should be combined with targeted training, such as increasing time spent outdoors, have shown promise in mitigating myopia progression and potentially influencing musculoskeletal health (63, 64). Strengthened early screening is crucial, prioritizing the identification of high-risk groups (e.g., females, students with heavy academic workloads) to mitigate the risk of comorbid myopia and scoliosis. Therefore, effective prevention and control necessitates collaboration among government, education, and health departments to enhance awareness among teachers and students regarding the hazards of poor posture, the importance of early intervention, and the need for regular screening. Integrating the aforementioned strategies into school health programs is essential. Building such an active and comprehensive integrated prevention system will effectively curb the onset and progression of both myopia and scoliosis, foster a healthier growth environment for adolescents, and yield significant synergistic benefits for both clinical practice and public health.

This study acknowledges several limitations. First, the identification of scoliosis relied solely on school-based screenings using scoliometer measurements, Precision limits of goniometers potentially underestimating minor postural deviations, future research should incorporate standardized radiographic assessments to enhance diagnostic precision. Second, while the focus was on reading/writing postures, influential factors such as outdoor activity duration and screen time were not included, potentially limiting the comprehensiveness of the risk factor analysis. Addressing these limitations requires future studies to broaden their scope, increase sample sizes, and incorporate additional relevant variables for a more holistic investigation of risk factors. Third, data on suboptimal reading/writing postures were derived from questionnaires rather than objective assessment tools; future studies could utilize home-based monitoring software and validated evaluation methods. Finally, longitudinal intervention studies are needed to rigorously evaluate the efficacy of the proposed multifaceted co-prevention strategies, such as those proposed in this study. This will be crucial for developing targeted, evidence-based preventive recommendations to promote adolescent health.

## Conclusions

Our findings indicate that poor postures are not only a risk factor for myopia and scoliosis individually but also exhibit a synergistic effect on comorbidity risk. Furthermore, a bidirectional relationship exists where each condition acts as a risk factor for the other, highlighting their complex interplay. These results emphasize the critical need for integrated approaches to prevent and manage myopia-scoliosis comorbidity, particularly among junior middle school students. Future research employing longitudinal designs, objective assessments, and diverse populations is warranted to confirm causality and explore effective interventions.

## Data Availability

The raw data supporting the conclusions of this article will be made available by the authors, without undue reservation.
